# *De Novo* Transcriptomes of a Mixotrophic and a Heterotrophic Ciliate from Marine Plankton

**DOI:** 10.1371/journal.pone.0101418

**Published:** 2014-07-01

**Authors:** Luciana F. Santoferrara, Stephanie Guida, Huan Zhang, George B. McManus

**Affiliations:** 1 Department of Marine Sciences, University of Connecticut, Groton, Connecticut, United States of America; 2 The National Center for Genome Resources, Santa Fe, New Mexico, United States of America; University of Cambridge, United Kingdom

## Abstract

Studying non-model organisms is crucial in the context of the current development of genomics and transcriptomics for both physiological experimentation and environmental characterization. We investigated the transcriptomes of two marine planktonic ciliates, the mixotrophic oligotrich *Strombidium rassoulzadegani* and the heterotrophic choreotrich *Strombidinopsis* sp., and their respective algal food using Illumina RNAseq. Our aim was to characterize the transcriptomes of these contrasting ciliates and to identify genes potentially involved in mixotrophy. We detected approximately 10,000 and 7,600 amino acid sequences for *S. rassoulzadegani* and *Strombidinopsis* sp., respectively. About half of these transcripts had significant BLASTP hits (E-value <10^−6^) against previously-characterized sequences, mostly from the model ciliate *Oxytricha trifallax*. Transcriptomes from both the mixotroph and the heterotroph species provided similar annotations for GO terms and KEGG pathways. Most of the identified genes were related to housekeeping activity and pathways such as the metabolism of carbohydrates, lipids, amino acids, nucleotides, and vitamins. Although *S. rassoulzadegani* can keep and use chloroplasts from its prey, we did not find genes clearly linked to chloroplast maintenance and functioning in the transcriptome of this ciliate. While chloroplasts are known sources of reactive oxygen species (ROS), we found the same complement of antioxidant pathways in both ciliates, except for one enzyme possibly linked to ascorbic acid recycling found exclusively in the mixotroph. Contrary to our expectations, we did not find qualitative differences in genes potentially related to mixotrophy. However, these transcriptomes will help to establish a basis for the evaluation of differential gene expression in oligotrichs and choreotrichs and experimental investigation of the costs and benefits of mixotrophy.

## Introduction

The most diverse and abundant ciliates in euphotic marine waters correspond to two sister subclasses, Oligotrichia and Choreotrichia (class Spirotrichea) [1]. Oligotrich and choreotrich ciliates are globally distributed [2] and episodically dominate microzooplankton [3]–[5]. They are major consumers of small algae, thus channeling energy through the microbial loop and higher levels in the planktonic food web [6]. One of the most prominent physiological differences between these two ciliate groups is that many oligotrich species practice mixotrophy, while this nutrition mode has not been confirmed for any choreotrich [7]–[10].

Mixotrophs obtain nutrients and energy by combining heterotrophy and autotrophy [11] and play key roles as both primary and secondary producers [12]. The mechanism for mixotrophy in oligotrichs is chloroplast sequestration, or kleptoplasty, in which a primarily herbivorous organism retains functional chloroplasts from its algal food and uses them for photosynthesis. For example, the oligotrich *Strombidium rassoulzadegani* captures chloroplasts from algal prey and uses them to grow rapidly in the light, although chloroplasts are not able to divide in the ciliate and eventually need to be replaced [13]–[16]. Apart from ciliates, kleptoplasty has been widely reported to occur within dinoflagellates, foraminiferans, and even in some molluscs [17]–[19].

It is unclear how a kleptoplastic organism can keep functional chloroplasts. Most genes needed to regulate these organelles are nuclear-encoded, but the algal nucleus is usually not retained in the host cell [10]. An exception is the ciliate *Mesodinium rubrum*, in which the nuclei of ingested algae remain transcriptionally active [20]. The most popular hypothesis on the genetic basis of kleptoplasty is related to the horizontal transfer of genes involved in chloroplast functioning and maintenance from algae to the host nucleus [21], [22]. For example, five plastid-targeting proteins that function in photosystem stabilization and metabolite transport have been found encoded in the nucleus of the kleptoplastic dinoflagellate *Dinophysis acuminata* and have probably been acquired through horizontal gene transfer from multiple algal sources [21]. In contrast, no support for horizontal gene transfer has been found in another kleptoplastic protist, the foraminiferan *Elphidium margaritaceum,* and thus other hypotheses related to chloroplast stability have been suggested [23], [24].

Kleptoplasty provides the advantage of a photosynthetic energy subsidy, but it is unclear if this strategy provides other benefits or costs to the cell [15]. One hypothetical cost of kleptoplasty is the necessity for mitigation of reactive oxygen species (ROS) produced during photosynthesis. ROS are produced during respiration and normal metabolism both in heterotrophic and autotrophic organisms, which have multiple mechanisms of detoxification [25], [26]. Additional ROS are produced and detoxified in the chloroplasts of autotrophs [27]–[29]. It is unknown how a kleptoplastic ciliate mitigates the extra ROS produced by the sequestered chloroplasts. Maintaining a different or more active detoxification mechanism and the risk of additional oxidative stress may represent costs of mixotrophy for ciliates. This is also interesting from the evolutionary point of view, as an enhanced ability to deal with ROS would partially explain why only some ciliates can harbor photosynthetic symbionts [30], [31]. On the other hand, some accumulation of ROS may provide defense against predation [32], thus helping to explain why mixotrophic ciliates appear to be less vulnerable than heterotrophic ones to copepod grazing [33].

The Marine Microbial Eukaryote Transcriptome Sequencing Project (MMETSP; http://marinemicroeukaryotes.org) has provided an unprecedented amount of genetic information on ciliates and other non-model marine protists that have been strongly underrepresented in previous genomics and transcriptomics efforts [34]. As part of this initiative, we performed RNAseq on two ciliates, the mixotrophic oligotrich *S. rassoulzadegani* and the heterotrophic choreotrich *Strombidinopsis* sp., and provide here the first transcriptome analyses for these groups. To eliminate potential food contamination, and given the lack of whole-genome information for any algae we could use as food, we also obtained the transcriptomes of the prey used for ciliate culturing. Our aim was to characterize the transcriptomes of these contrasting ciliates and, in particular, to explore the hypothesis that additional genes involved in kleptoplasty and ROS mitigation are expressed by the mixotroph compared to the heterotroph. Given the complete novelty of this kind of data, our study advances the understanding of the physiology of mixotrophic and heterotrophic ciliates and lays the molecular groundwork necessary for further experimentation.

## Materials and Methods

### Ethics statement

*Strombidium rassoulzadegani* and *Strombidinopsis* sp. were sampled from a tide pool and a dock, respectively, at the UConn Avery Point campus, Connecticut, USA (41.32° N, 72.06° W). No special permits were needed and field collection did not involve endangered or protected species.

### Cultures and RNA extraction

Both ciliates were isolated and maintained in autoclaved, filtered seawater supplemented with mineral nutrients (salinity c. 30 Practical Salinity Units and nutrients added as f/2 or f/20 for the mixotroph and the heterotroph, respectively [35]). *S. rassoulzadegani* has been kept in our laboratory for almost 10 years using the prasinophyte *Tetraselmis chuii* (strain PLY429), which is the prey that provides the most efficient growth of this ciliate [15]. *Strombidinopsis* sp. has been periodically isolated and cultured in our laboratory using the cryptophyte *Rhodomonas lens* (strain RHODO) as food. For RNA isolation, new cultures of *S. rassoulzadegani* and *Strombidinopsis* sp. were started using *T. chui* PLY429 and *R. lens* RHODO, respectively, as prey.

When the ciliate cultures were in the exponential growth stage and the food algae in the culture were largely consumed, ciliate cells were harvested. To minimize food contamination in the ciliate RNA extracts, individual cells were picked with a micropipette under a stereo microscope and pooled into a 15-ml tube containing 5 ml of Tri-Reagent (MRC Inc., Cincinnati, OH, USA). A total of 22,000 cells for *S. rassoulzadegani* and 10,000 for *Strombidinopsis* sp. were isolated. Also, the two food algae (∼10^7^ cells) were harvested from axenic cultures by centrifugation at 3,000 x g and the cell pellets were fixed in Tri-Reagent. RNA was extracted from all four samples following the modified Zymo column purification method using Direct-zol RNA MiniPrep Kit (Zymo Research, Irvine, CA, USA) as reported previously [36].

### Library preparation and RNAseq

RNA samples were quantified using Qubit Q32855 (Invitrogen, Carlsbad, CA, USA) and their quality was assessed using the Agilent 2100 Bioanalyzer. Libraries for each of the four species were made from 2 µg RNA using the TruSeq RNA Sample Preparation Kit (Illumina, San Diego, CA, USA). Libraries were sequenced on the Illumina HiSeq 2000 to obtain paired-end, 50-bp-long reads. Approximately 2 Gbp of sequence data was generated per library.

### Assembly

Transcriptome assembly was carried out using the internal pipeline BPA1.0 (Batch Parallel Assembly version 1.0) of the National Center for Genome Resources. Sequence reads were preprocessed using SGA [37] for quality trimming (swinging average) at Q15. Reads shorter than 25 nucleotides (nt) after trimming were discarded. Preprocessed sequence reads were assembled into contigs with ABySS v. 1.3.0 [38], using 20 unique kmers between k = 26 and k = 50. ABySS was run requiring a minimum kmer coverage of 5, and bubble popping at >0.9 branch identity with the scaffolding flag enabled to maintain contiguity for divergent branching. Paired-end scaffolding was performed on each kmer. Sequence read pairing information was used in GapCloser v. 1.10 as part of the SOAP *de novo* package [39] to walk in on gaps created during scaffolding in each individual kmer assembly. Contigs from all gap-closed kmer assemblies were combined. The OLC (overlap layout consensus) assembler miraEST [40] was used to identify minimum 100 base pair overlaps between the contigs and assemble larger contigs, while collapsing redundancies. BWA [41] was used to align sequence reads back to the contigs. Alignments were processed by SAMtools mpileup (http://samtools.sourceforge.net) to generate consensus nucleotide calls at positions where IUPAC bases were introduced by miraEST [40], and read composition showed a predominance of a single base. In an attempt to remove incomplete sequences, the consensus contigs were filtered at a minimum length of 150 nt to produce the final set of contigs. Sequences are available in the CAMERA Portal (http://camera.calit2.net/mmetsp/list.php [42]) under the unique MMETSP identifiers included in Table 1.

**Table 1:** Transcriptome statistics.

Species	*Strombidium rassoulzadegani*	*Tetraselmis chuii*	*Strombidinopsis* sp.	*Rhodomonas lens*
**Sample identifier**	MMETSP0449	MMETSP0491	MMETSP0463	MMETSP0484
**Illumina pair-end reads**	24,756,222	13,857,343	43,171,474	21,891,882
**Number of contigs**	12,163	26,975	24,981	33,177
**Number of characters**	12,354,690	33,029,773	29,714,767	36,097,100
**Contig maximum length**	6,635	16,863	14,021	18,919
**Contig N50**	1,307	1,770	1,589	1,519
**Reads realigned to contigs**	90%	83%	89%	87%
**Predicted CDS**	10,562	22,551	8,619	30,293
**Predicted AAS**	10,825	23,036	9,674	30,802
**Food filtered CDS**	9,752	-	6,553	-
**Food filtered AAS**	10,015	-	7,608	-

CDS  =  DNA coding sequences; AAS  =  amino acid sequences.

### Prediction of coding regions and elimination of food transcripts

DNA coding sequences (CDS) and the corresponding amino acid sequences (AAS) were predicted using ESTScan [43], [44]. Resulting CDS and AAS numbers were slightly different given the different length cut-offs used for each dataset (150 nt vs. 30 aa, respectively). A Bacillariophyta scoring matrix was used based on availability of well-annotated mRNA entries in NCBI RefSeq. Illumina sequence reads were aligned back to the nucleotide motifs of the assembled contigs and predicted CDS using BWA [41] to assess assembly quality.

Putative algal sequences in the ciliate data were identified with BLASTN [45] using the food algae transcripts as reference database and an E-value of 10^−6^ as cut-off. Sequences with a significant hit were removed from the ciliate datasets using custom scripts.

### Sequence homology

Ciliate AAS datasets were contrasted with OrthoMCL using default settings [46]. First, all-against-all BLASTP searches (E-value <10^−6^) were done to identify reciprocal best hits between species. Then, homologous AAS were grouped and each group was putatively classified as orthologous (gene families separated by speciation) or paralogous (gene duplications subsequent to speciation).

### Annotation

Predicted AAS were annotated using Blast2GO [47]. The NCBI non-redundant NR database and an E-value cut-off of 10^−6^ were used for BLASTP [45]. Annotated Gene Ontology (GO) terms were complemented with results from InterProScan and associated KEGG pathways were retrieved. In addition to Blast2GO, AAS characterization was done also with the more sensitive method HMMER3 [48] against the Pfam-A [49], TIGRFAM [50] and SUPERFAMILY [51] databases. Information on proteins of particular interest (e.g. related to photosynthesis or response to ROS) was retrieved manually from both Blast2GO and HMMER3 results. This strategy was used also to confirm absence of certain AAS in *Strombidinopsis* sp. or algae datasets.

### Phylogenetic inferences

For one protein of interest (Nec3, see below), ciliate transcripts and other amino acid sequences downloaded from NCBI GenBank were combined and aligned with MUSCLE [52]. Overlapping regions were trimmed resulting in a final alignment of 225 sites. For phylogenetic inferences, both Neighbor Joining (as implemented in MEGA [53]) and Maximum Likelihood (RAxML [54]) analyses were carried on with 1,000 bootstrap replicates. The evolution model LG with a Г model of rate heterogeneity and a proportion of invariable sites was used as selected by ProtTest under the Akaike Information Criterion [55].

## Results and Discussion

### Transcriptome assemblies, filtering of food transcripts, and ciliate AAS datasets

We sequenced the transcriptomes of two marine planktonic ciliates, *Strombidium rassoulzadegani* and *Strombidinopsis* sp., as well as their two respective algal foods. The number of Illumina reads and assembled contigs obtained in this study ranged from ca. 14 to 43 million and 12 to 33 thousand per species, respectively (Table 1). Half of the total assembled nucleotides were contained in sequences of 1,300 nt or larger as indicated by N50 values (minimum size cut-off  =  150 nt). The fact that over 80% of Illumina reads were realigned to these contigs confirms the adequate quality of the assemblies. However, there are no reference genomes for any of the ciliates and algae sequenced and thus we cannot make any conclusions about the completeness of the transcriptomes. For ciliates, the closest species with a known genome is *Oxytricha trifallax*, which belongs to a different subclass (Stichotrichia) of the Spirotrichea. The genome of this species, which is fragmented into thousands of nanochromosomes, is about 50 Mb long and is estimated to encode ca. 18,400 genes [56]. This gene content is within the range of transcripts assembled for *S. rassoulzadegani* and *Strombidinopsis* sp. (Table 1), although it is unclear how comparable the *O. trifallax* genome is to those of Oligotrichia and Choreotrichia species.

**Figure 1:**
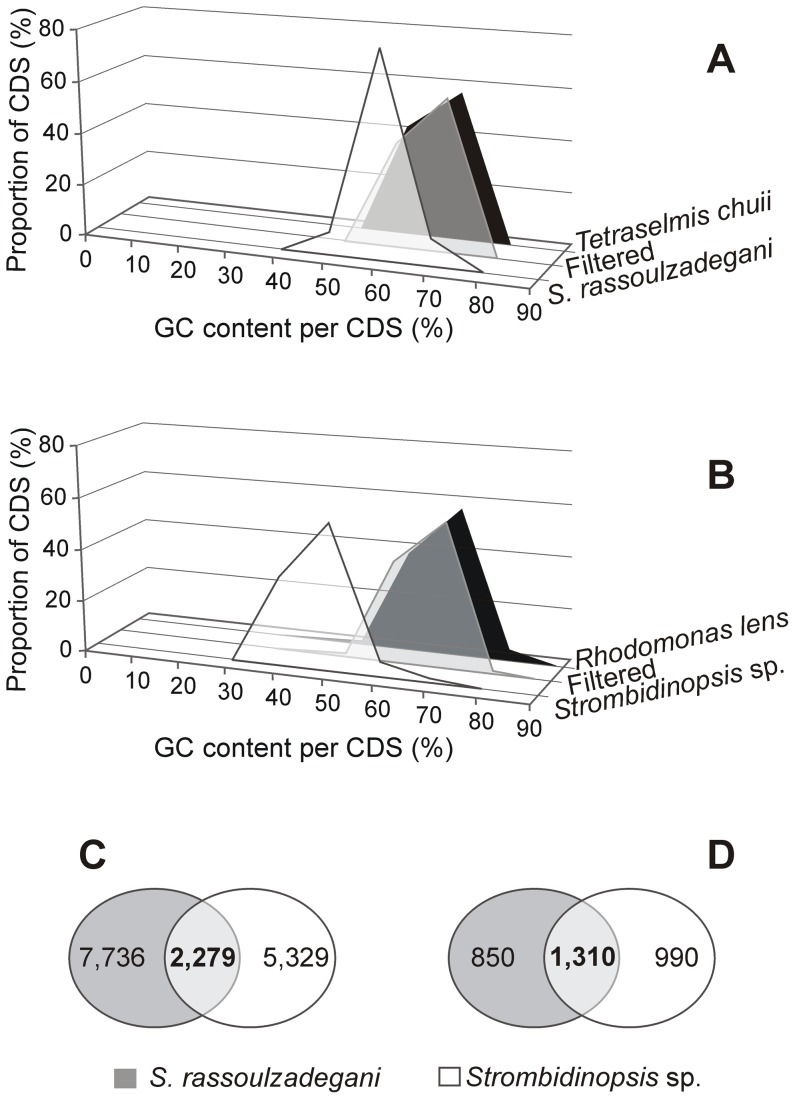
Comparison of *Strombidium rassoulzadegani* and *Strombidinopsis* sp. transcriptomes. A-B Frequency distribution of GC content in ciliate transcripts, algal food transcripts, and putative food transcripts filtered from ciliate data. C- Venn diagram of all-against-all BLASTP hits between ciliates. D- Venn diagram of orthologous and paralogous amino acid sequence groups in ciliates.

Given that oligotrich and choreotrich ciliates cannot be cultured independently of their food, we had to include a step to eliminate putative algal transcripts from the ciliate data. Although ciliate cells were picked individually to avoid contamination, algal 18S rRNA was detected in the ciliate samples, possibly due to prey being digested within the ciliates. A total of 7.7% and 24.0% of the CDS (equivalent to 5% and 6% of Illumina reads) obtained from *S. rassoulzadegani* and *Strombidinopsis* sp. cultures, respectively, were removed as food transcripts. Analysis of GC content in CDS indicated that the filtering procedure was successful (Fig. 1A-B). The frequency distribution of GC content per CDS was identical between algal transcripts and transcripts eliminated from ciliate data. In contrast, the distribution of filtered ciliate transcripts showed distinctive peaks, with maximum frequency of CDS with 60% and 40–50% GC content in *S. rassoulzadegani* and *Strombidinopsis* sp., respectively.

We detected 10,015 AAS for *S. rassoulzadegani* and 7,608 AAS for *Strombidinopsis* sp. (Table 1). Using OrthoMCL, 2,279 out of the total 17,623 AAS were identified as reciprocal best hits between the two species (Fig. 1C). In addition, 1,310 out of 3,150 total AAS groups were shared between species (orthologous), while the remaining groups were identified as paralogs within *S. rassoulzadegani* or *Strombidinopsis* sp. (Fig. 1D). Thus, our transcriptome data indicated only 13% reciprocal best hits pairs and 42% orthologous groups between the two ciliates.

### *Strombidium rassoulzadegani* and *Strombidinopsis* sp. transcriptome annotation

**Figure 2:**
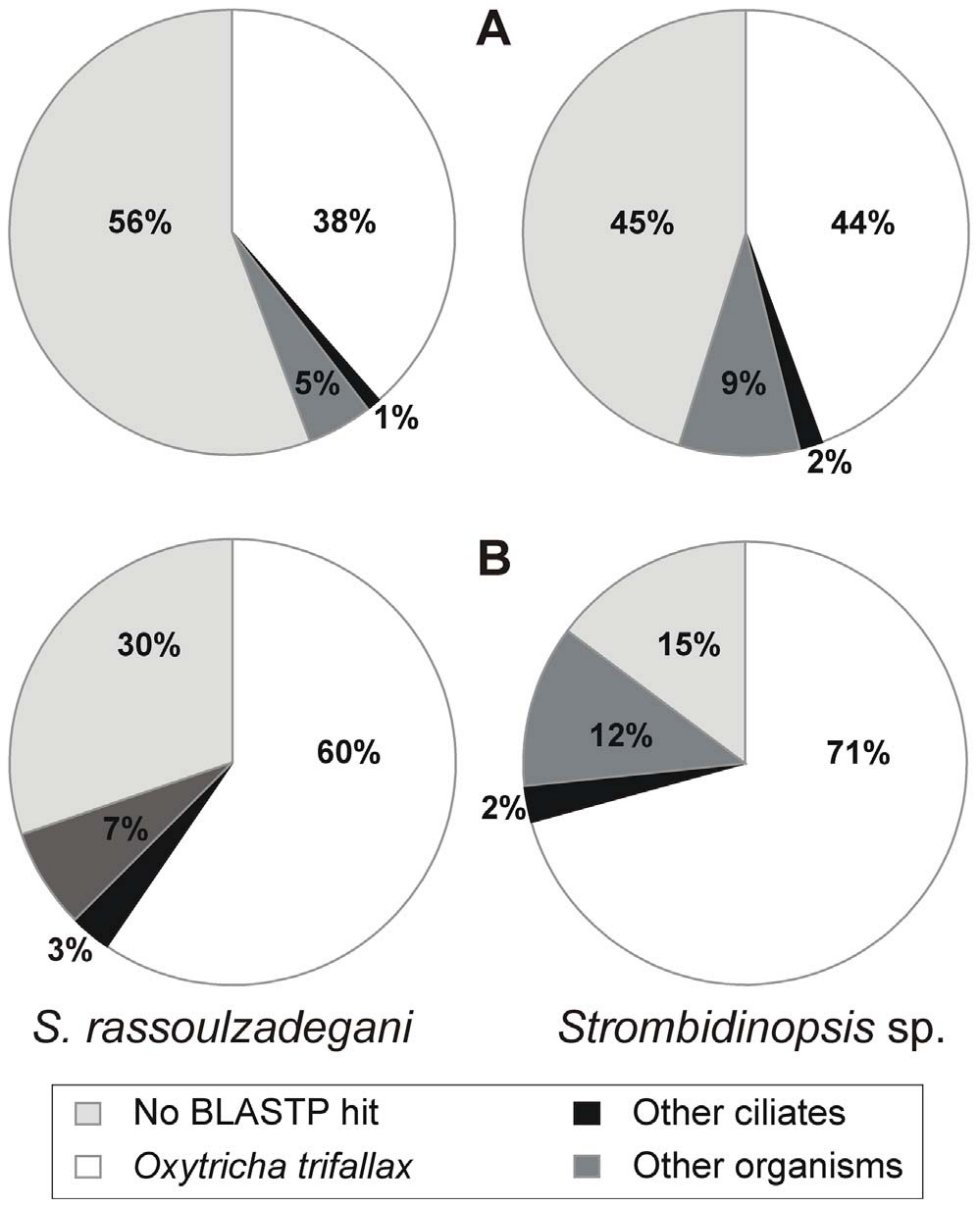
BLASTP hit distribution in ciliate transcripts. A- Proportion of amino acid sequences. B- Proportion of Illumina reads realigned to the corresponding coding regions.

About half of *Strombidium rassoulzadegani* and *Strombidinopsis* sp. transcripts matched previously known sequences. A total of 44% and 55% predicted AAS (equivalent to 70% and 85% Illumina reads) had significant BLASTP hits (NCBI non redundant NR database, E-value <10^−6^) for *S. rassoulzadegani* and *Strombidinopsis* sp., respectively (Fig. 2). In both cases, the maximum proportion of hits corresponded to *Oxytricha trifallax*. Only eight ciliate genomes have been sequenced so far [57], thus explaining the proportion of unknown sequences. However, this proportion is relatively low in comparison to that found for other non-model protist transcriptomes (e.g. 72% unknown sequences for a marine euglenoid [58]). From the low proportion of our AAS that matched to sequences from groups other than ciliates, most of them corresponded to lineages such as amoebozoa and opisthokonts. Less than 0.5% of hits corresponded to the same lineages as the food algae (prasinophytes or cryptophytes).

**Figure 3:**
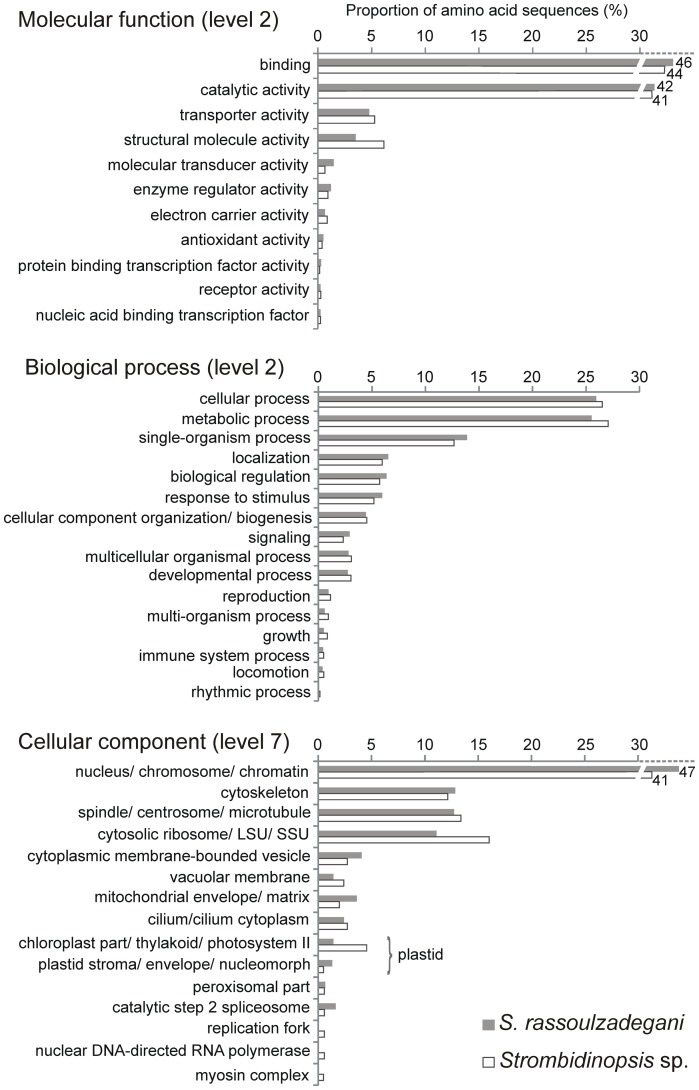
Gene Ontology terms distribution in ciliate transcripts.

From the AAS with significant BLASTP hits, 40% (*S. rassoulzadegani*) and 47% (*Strombidinopsis* sp.) had a confident assignment of Gene Ontology (GO) terms, which were retrieved mostly from the UniProt database. Complementation with InterProScan results increased annotations by 19% (*S. rassoulzadegani*) and 22% (*Strombidinopsis* sp.). GO terms distribution was similar between species (Fig. 3), with binding and catalytic activity as the main molecular functions, cellular and metabolic process as the main biological processes and nuclear-related structures as the main cellular components represented in both transcriptomes.

For each ciliate dataset, transcripts were included in 92 KEGG pathways, 81 of which were present in both species (Table S1). Most of these pathways corresponded to the metabolism of carbohydrates, lipids, amino acids, nucleotides, glycan, terpenoids, and vitamins and cofactors. In addition, some sequences were related to biosynthesis of secondary metabolites such as some antibiotics (e.g. streptomycin, neomycin) and degradation of xenobiotics such as some toxic aromatic hydrocarbons (e.g. xylene, toluene). A few of the results obtained by automatic annotations with Blast2GO were unexpected for protists. Some GO terms (e.g. ‘multicellular organismal process’ or ‘immune system process’; Fig. 3) may actually represent ancient eukaryotic genes with broader functions [59]. Similar conclusions may apply for some KEGG pathways linked to both ciliate datasets (e.g. ‘peptidoglycan biosynthesis’; Table S1).

### Transcripts potentially related to kleptoplasty in *Strombidium rassoulzadegani*

A kleptoplastic organism engulfs photosynthetic prey and digests all but their chloroplasts, which remain temporarily functional despite lacking control from the algal nucleus. Some kleptoplastic organisms express algal genes involved in chloroplast functioning and photosynthesis, likely integrated in the host nucleus by horizontal gene transfer [21], [22]. We found transcripts linked to the GO term ‘plastid’ and the KEGG pathway ‘carbon fixation in photosynthetic organisms’ in the kleptoplastic *Strombidium rassoulzadegani*, but this was detected in the heterotrophic *Strombidinopsis* sp. as well (Fig. 3; Table S1). Specific searches of transcripts assigned to GO terms ‘plastid’ and ‘photosynthesis’ in both ciliates (Table S2) indicated that most of those transcripts 1) had a significant BLASTP hit with *O. trifallax* or other non-photosynthetic organisms and/or 2) are not clearly specific to plastids or photosynthesis according to their GO terms descriptions and associated KEGG pathways. Thus, most of these transcripts probably have more general functions. Apart from not providing data on chloroplast functioning in *S. rassoulzadegani*, some of these sequences may correspond to the <0.5% potential food transcripts not filtered from the ciliate data (see above), especially the few of them that had significant BLASTP hits with the same lineages as the algal prey (Table S2).

Alternative explanations for kleptoplasty include that retained chloroplasts are simply stable and thus remain functional for some time [23], [24] or that they are transcriptionally active and can regulate themselves inside the host. The methods used in this study prevent us from discriminating if chloroplast genes from the food alga are expressed in *S. rassoulzadegani*. If this is the case, chloroplast genes expressed within the ciliate may have been removed by poly-A selection during library preparation and/or by filtering sequences that matched with algal transcripts in BLASTN searches. Similar to our results, transcriptome data on a kleptoplastic foraminiferan were also unable to provide information on genes potentially related to chloroplast functioning in the host [24]. Thus, this approach may be insufficient to explain the mechanics of kleptoplasty in some organisms.

### ROS detoxification in *Strombidium rassoulzadegani* and *Strombidinopsis* sp

**Figure 4:**
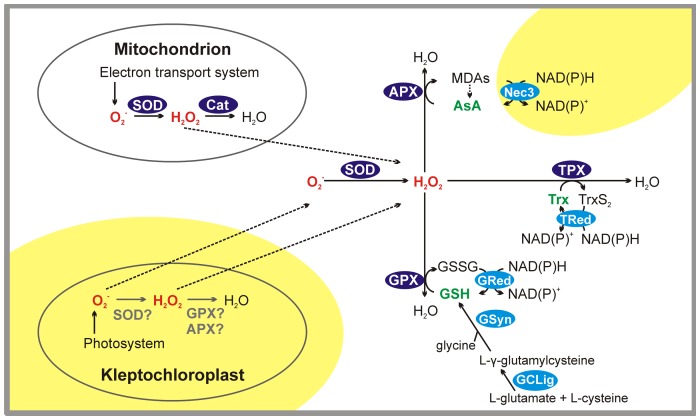
Hypothetical pathways for reactive oxygen species (ROS) detoxification. Pathways evidenced in both ciliates (white background) or only in *Strombidium rassoulzadegani* (yellow background) are shown. In red, ROS (O_2_^-^  =  superoxide radical; H_2_O_2_  =  hydrogen peroxide); in dark blue, antioxidant enzymes (SOD  =  superoxide dismutase; Cat  =  catalase; APX  =  ascorbate peroxidase; GPX  =  glutathione peroxidase; TPX  =  thioredoxin peroxidase); in light blue, enzymes involved in metabolism of non-enzymatic antioxidants (Nec3  =  bifunctional monodehydroascorbate reductase/carbonic anhydrase nectarin-3-like; TRed  =  thioredoxin reductase; GRed  =  glutathione reductase; GSyn  =  GSH synthetase; GCLig  =  Glutamate-cysteine ligase); in green, non-enzymatic antioxidants (AsA  =  ascorbic acid; GSH  =  glutathione; Trx  =  thioredoxin). MDAs  =  monodehydroascorbate; GSSG  =  glutathione disulfide; TrxS_2_  =  thioredoxin disulfide; NAD(P)^+^  =  nicotinamide adenine dinucleotide (phosphate). Based on literature [61], [63], [75].

Both under normal physiological conditions and as a response to oxidative stress, autotrophic and heterotrophic cells have enzymatic and non-enzymatic mechanisms to control ROS concentrations [25], [26], [28]. We found evidence for these pathways in the transcriptomes of *Strombidium rassoulzadegani* and *Strombidinopsis* sp. (Fig. 4, Tables S3 and S4). In this case, transcripts were clearly linked to known antioxidant enzymes and most of them had highly significant BLASTP hits against ciliates or other non-photosynthetic organisms, thus minimizing the possibility that these genes belong to the food algae (Tables S3 and S4). Although we show simplified antioxidant pathways, these mechanisms are usually interrelated by reciprocal control and each of them has a higher activity in a certain cell compartment or against a certain oxidant, including ROS, lipid peroxides and reactive nitrogen species [29].

Superoxide dismutase, catalase and peroxidases, the major enzymes that directly modulate ROS, were detected in the transcriptomes of both *S. rassoulzadegani* and *Strombidinopsis* sp. (Fig. 4, Table S3). Superoxide dismutase (SOD) reduces superoxide radicals to hydrogen peroxide and it exists in two forms in both ciliates: Cu/Zn SOD and Fe/Mn SOD (cytosolic and mitochondrial forms, respectively, in higher eukaryotes [60]). Catalase and peroxidases reduce hydrogen peroxide to water, the latter using non-enzymatic antioxidants as electron donors [25]. Ascorbate peroxidase (APX), glutathione peroxidase (GPX) and thioredoxin peroxidase (TPX) oxidize ascorbic acid (AsA), glutathione (GSH) and thioredoxin (Trx), respectively, in order to reduce hydrogen peroxide [61], [62]. Catalase, APX, GPX and TPX sequences were found in both ciliates. The multiple sequences detected for each enzyme may correspond to isoforms with different cell localizations, as known for example for APX and GPX in algae and plants [63]. They may also correspond to mRNA precursors, which are usually difficult to distinguish in RNAseq data [64]. An additional cause for these multiple sequences may be inability to condense some similar transcripts during the assembly.

Among non-enzymatic antioxidants, GSH is a tri-peptide that can be synthetized and recycled in both ciliates, according to the transcriptome data (Fig. 4, Table S4). Similarly, transcripts for the protein Trx and its recycling enzyme were found in both species. In contrast, transcripts related to the synthesis of AsA or other antioxidants found in plants and algae, such as carotenoids and tocopherols [65], were not detected in the ciliates.

Interestingly, we found evidence for a group of enzymes that can recycle AsA (nectarin-3-like enzymes, Nec3) in *S. rassoulzadegani* but not in *Strombidinopsis* sp. (Fig. 4, Table S4). Nec3 has monodehydroascorbate reductase (MDAR) activity, i.e. it transforms the oxidized form of AsA back to its reduced form, thus providing the advantage of keeping constant levels of AsA without the necessity of a constant supply [66]–[70]. Although AsA can also be recycled spontaneously or through a cycle that involves GSH (the AsA-GSH cycle), it is more rapidly regenerated by MDAR activity [29]. Therefore, Nec3 may help *S. rassoulzadegani* to maintain high pools of AsA, which is important both as anti-oxidant and as cofactor and regulator during photosynthesis [29]. Phylogenetic inferences showed that Nec3 from *S. rassoulzadegani* clustered with sequences from other non-photosynthetic organisms (the ciliate *Oxytricha trifallax*, one fungus and two animals) and formed a group apart from those of plants, although there are no sequences available for algae (Fig. S1). These preliminary results suggest that 1) Nec3 belongs to the ciliate and not the food alga, and 2) there is no evolutionary link between *S. rassoulzadegani* and photosynthetic organisms regarding Nec3. In this context, clarifying the origin and role of Nec3 in *S. rassoulzadegani* and its potential role in mixotrophy deserves further experimentation.

ROS detoxification occurs in several parts of the eukaryotic cell. In both heterotrophic and autotrophic cells these mechanisms act in the cytosol, in mitochondria and, in many eukaryotes, also in peroxisomes. Peroxisome presence has a patchy distribution among ciliates and other protist taxa [71] and they have not been observed in oligotrichs or choreotrichs to our knowledge, but we detected sequences related to this organelle in our data (Fig. 3). In autotrophs, enzymes such as SOD, APX and GPX scavenge ROS also in the chloroplast and they are key in order to keep this organelle active [29], [63], [72]. However, these enzymes are nuclear encoded [60], [73] and, even if they initially exist in kleptochloroplasts of *S. rassoulzadegani*, their activity is very likely lost after some time (Fig. 4). Inactivation of chloroplast antioxidant enzymes is known to limit photosynthetic efficiency [74]. Thus, oxidative damage may contribute to the lack of kleptochloroplast functionality and the fact that a continuous supply of fresh chloroplasts is needed for the growth of *S. rassoulzadegani* [15].

## Conclusions

We used RNAseq to characterize the transcriptomes of two non-model microbial eukaryotes. This approach provided information about genes with known functions as well as multiple potentially novel genes. We experienced the typical challenges of studying non-model organisms and using automatic annotation tools that do not detect the whole spectrum of protist physiological features. Limitations such as unknown levels of genome coverage, high proportion of sequences not similar to those available in databases, and annotations not compatible with protist biology have been common in this kind of study so far. Additional effort was required for sequencing and filtering food transcripts, given that the ciliates under study cannot be cultured independent of their prey.

The transcriptomes of *Strombidium rassoulzadegani* and *Strombidinopsis* sp. provide baselines for analyzing ciliate metabolism, ecological roles in the planktonic food web and relationships with the environment. Our observations are noteworthy in two ways. First, we analyzed the first transcriptomes from oligotrichs and choreotrichs, which are the most diverse and abundant ciliates in marine plankton. Second, the species we chose practice two contrasting nutritional modes, heterotrophy and mixotrophy, and hence have somewhat different ecological roles. Although the transcriptomes differed in general features such as GC content distribution and had a homology lower than 50%, they provided similar annotations for GO terms and KEGG pathways, which were related mostly to housekeeping activity. As more ciliate reference genomes become available, we expect that more pathways, including novel ones, will be revealed in the data.

Transcriptome information alone provided limited insights on genes related to mixotrophy. We did not find transcripts clearly related to the maintenance and functioning of retained chloroplasts in *S. rassoulzadegani* and we identified very similar antioxidant mechanisms in both mixotrophic and heterotrophic ciliates. The relevance of one enzyme potentially related to ascorbate recycling in the mixotroph as well as the potential differences in regulation and expression levels of all the identified genes require future experimentation in order to understand the implications of antioxidant pathways for physiology and evolution of mixotrophs.

## Supporting Information

Figure S1 Maximum Likelihood tree inferred from Nec3 sequences. Nodes with support higher than 50% are indicated with a black (Maximum Likelihood) and/or a grey circle (Neighbor Joining). The scale bar represents ten substitutions per 100 nucleotides.
(TIF)

Table S1 KEGG pathways identified in *Strombidium rassoulzadegani* and *Strombidinopsis* sp.
(XLSX)

Table S2 Results of specific searches for GO terms related to plastids and photosynthesis in *Strombidium rassoulzadegani* and *Strombidinopsis* sp. transcriptomes.
(XLSX)

Table S3 Transcripts of antioxidant enzymes detected in *Strombidium rassoulzadegani* and *Strombidinopsis* sp.
(DOCX)

Table S4 Transcripts related to metabolism of non-enzymatic antioxidants in *Strombidium rassoulzadegani* and *Strombidinopsis* sp.
(DOCX)
