# Supplementary figures and images for: De Novo Transcriptomes of a Mixotrophic and a Heterotrophic Ciliate from Marine Plankton
Source: PLoS One. 2014 Jul 1;9(7):e101418. doi: 10.1371/journal.pone.0101418 (PMC4077812; doi:10.1371/journal.pone.0101418)

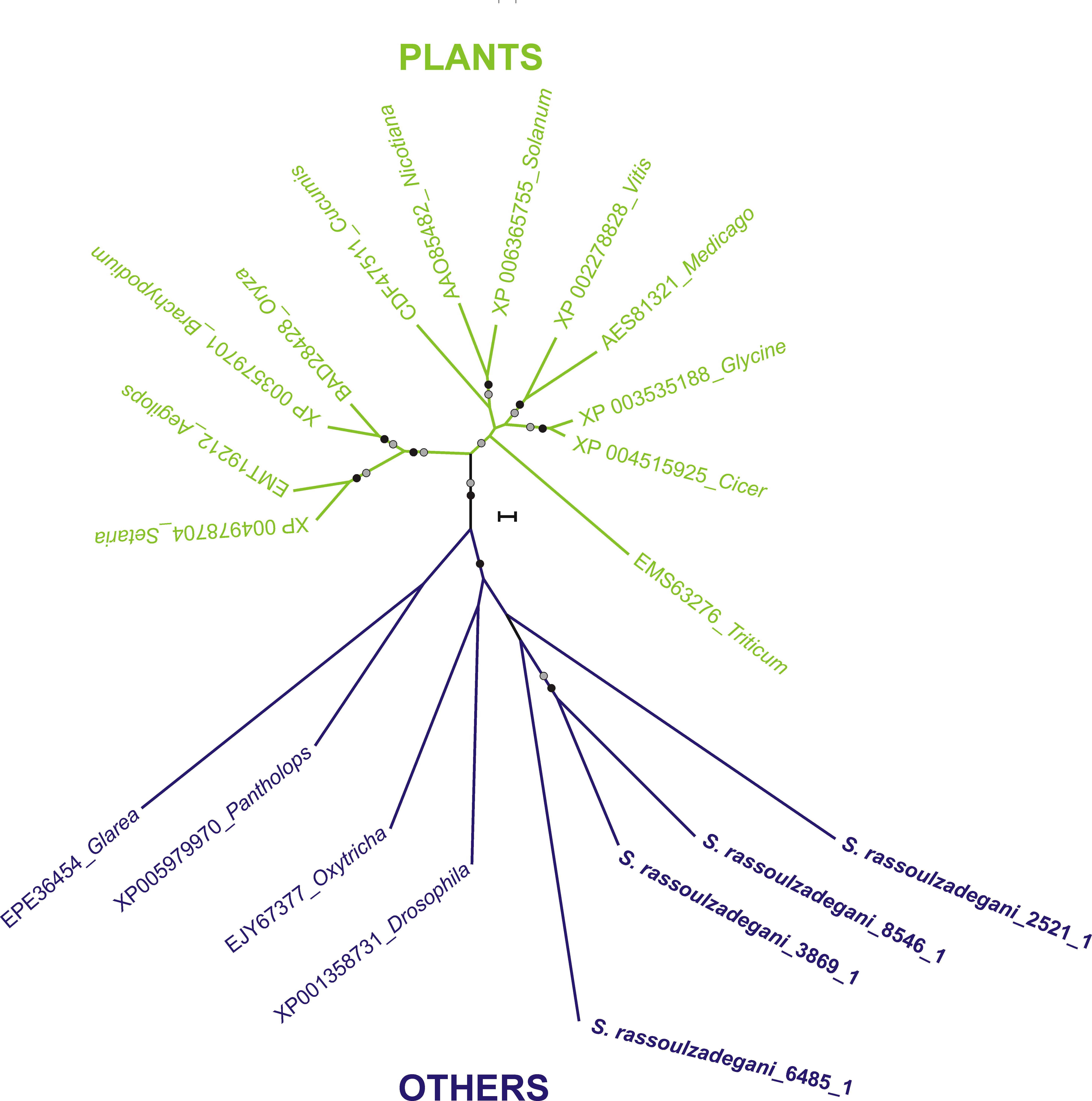

Supplement: Figure S1 — Maximum Likelihood tree inferred from Nec3 sequences. Nodes with support higher than 50% are indicated with a black (Maximum Likelihood) and/or a grey circle (Neighbor Joining). The scale bar represents ten substitutions per 100 nucleotides. (TIF) [file pone.0101418.s001.tif]
