# Supplementary material for: De Novo Transcriptomes of a Mixotrophic and a Heterotrophic Ciliate from Marine Plankton
Source: PLoS One. 2014 Jul 1;9(7):e101418. doi: 10.1371/journal.pone.0101418 (PMC4077812; doi:10.1371/journal.pone.0101418)
Supplement: Table S3 — Transcripts of antioxidant enzymes detected in Strombidium rassoulzadegani and Strombidinopsis sp. (DOCX) [file pone.0101418.s004.docx]

| **Sequence Name** | **Amino acids** | **Top Blast Hit** | **ACC** | **E-Value** |
| --- | --- | --- | --- | --- |
| **Superoxide dismutase EC:1.15.1.1**  (196 amino acids, evidence at protein level for *Tetrahymena pyriformis*) | | | | |
| Cu/Zn superoxide dismutase | | | | |
| Sras_3193_1 | 162 | *Ichthyophthirius multifiliis* (Ciliate) | XP_004035843 | 1.53E-63 |
| Sras_8231_1 | 215 | *Oxytricha trifallax* (Ciliate) | EJY70280 | 1.13E-16 |
| Sras_8945_1 | 196 | *Ruditapes philippinarum* (Animal) | ACU83236 | 1.04E-52 |
| Sopsis_8586_1 | 109 | *Oxytricha trifallax* (Ciliate) | EJY71389 | 1.26E-40 |
| Sopsis_9836_1 | 214 | *Cryptocaryon irritans* (Animal) | AEG91001 | 2.77E-13 |
| Sopsis_10394_1 | 86 | *Trichoderma atroviride* (Fungus) | EHK47440 | 1.50E-29 |
| Sopsis_13681_1 | 212 | *Ceratotherium simum simum* (Animal) | XP_004429611 | 6.92E-15 |
| Fe/Mn superoxide dismutase | | | | |
| Sras_729_1 | 216 | *Oxytricha trifallax* (Ciliate) | EJY64509 | 1.13E-54 |
| Sras_613_1 | 283 | *Oxytricha trifallax* (Ciliate) | EJY66799 | 7.73E-72 |
| Sras_8073_1 | 111 | *Lactuca sativa* (Plant) | CAC69402 | 9.92E-13 |
| Sopsis_10352_1 | 215 | *Oxytricha trifallax* (Ciliate) | EJY64509 | 4.07E-62 |
| Sopsis_13576_1 | 202 | *Salpingoeca sp.* (Opisthokonta) | XP_004990675 | 7.09E-74 |
| Sopsis_7996_1 | 239 | *Salpingoeca sp.* (Opisthokonta) | XP_004990675 | 1.04E-71 |
| **Catalase EC:1.11.1.6**  (487 amino acids, evidence at protein level for *Candida albicans*; 470 amino acids, inferred from homology for *Paramecium bursaria*) | | | | |
| Sras_3125_1 | 475 | *Oxytricha trifallax* (Ciliate) | EJY78794 | 0 |
| Sras_10397_1 | 98 | *Capsaspora owczarzaki* (Opisthokonta) | XP_004364341 | 1.30E-46 |
| Sras_1039_1 | 108 | *Azospirillum brasilense* (Bacteria) | YP_004987249 | 6.99E-44 |
| Sopsis_8719_1 | 465 | *Oxytricha trifallax* (Ciliate) | EJY78794 | 0 |
| **Ascorbate peroxidase EC:1.11.1.11**  (282 amino acids, evidence at protein level for *Chlamydomonas* sp.; 273 amino acids, inferred from homology for *Oxythicha trifallax* ) | | | | |
| Sras_11695_1 | 275 | *Oxytricha trifallax* (Ciliate) | EJY74724 | 6.63E-37 |
| Sras_5529_1 | 346 | *Oxytricha trifallax* (Ciliate) | EJY86286 | 8.87E-64 |
| Sras_8984_1 | 500 | *Amphidinium carterae* (Dinoflagellate) | ACF28633 | 1.52E-34 |
| Sopsis_8286_1 | 118 | *Oxytricha trifallax* (Ciliate) | EJY86286 | 2.41E-20 |
| Sopsis_13555_1 | 158 | *Oxytricha trifallax* (Ciliate) | EJY74724 | 4.98E-29 |
| **Glutathione peroxidase EC:1.11.1.9**  (184 amino acids, evidence at transcript level for *Moneuplotes crassus*) | | | | |
| Sras_6347_1 | 161 | *Tetrahymena thermophile* (Ciliate) | XP_001022734 | 5.23E-41 |
| Sras_3272_1 | 202 | *Oxytricha trifallax* (Ciliate) | EJY83199 | 2.21E-39 |
| Sras_5907_1 | 176 | *Aerococcus viridans* (Bacteria) | WP_016896637 | 1.92E-13 |
| Sopsis_11273_1 | 177 | *Moneuplotes crassus* (Ciliate) | ACL81236 | 2.82E-22 |
| **Thioredoxin peroxidase EC:1.11.1.15**  (196 amino acids, evidence at transcript level for *Candida albicans*) | | | | |
| Sras_3001_1 | 164 | *Oxytricha trifallax* (Ciliate) | EJY85734 | 3.50E-70 |
| Sras_6146_1 | 185 | *Trypanosoma cruzi* (Euglenozoa) | CAA06923 | 2.92E-64 |
| Sopsis_8114_1 | 222 | *Oxytricha trifallax* (Ciliate) | EJY71738 | 4.62E-80 |
| Sopsis_11137_1 | 117 | *Oxytricha trifallax* (Ciliate) | EJY73729 | 3.55E-50 |

**Table S3. Transcripts of antioxidant enzymes detected in *Strombidium rassoulzadegani* (Sras) and *Strombidinopsis* sp. (Sopsis).** Enzyme Codes (EC) and expected sequence length are provided for each enzyme.
