# Supplementary material for: De Novo Transcriptomes of a Mixotrophic and a Heterotrophic Ciliate from Marine Plankton
Source: PLoS One. 2014 Jul 1;9(7):e101418. doi: 10.1371/journal.pone.0101418 (PMC4077812; doi:10.1371/journal.pone.0101418)
Supplement: Table S4 — Transcripts related to metabolism of non-enzymatic antioxidants in Strombidium rassoulzadegani and Strombidinopsis sp. (DOCX) [file pone.0101418.s005.docx]

| **Sequence Name** | **Amino acids** | **Description** | **Top Blast Hit** | **ACC** | **E-Value** | **EC** |
| --- | --- | --- | --- | --- | --- | --- |
| **Metabolism of glutathione** | | | | | | |
| Sras_11551_1 | 452 | Glutathione reductase | *Oxytricha trifallax*  (Ciliate) | EJY84943 | 6.76E-164 | 1.8.1.7 |
| Sras_1478_1 | 451 | Glutathione reductase | *Oxytricha trifallax* | EJY84943 | 8.08E-166 | 1.8.1.8 |
| Sopsis_4076_1 | 204 | Glutathione reductase | *Oxytricha trifallax* | EJY87957 | 3.38E-67 | 1.8.1.9 |
| Sopsis_10346_1 | 481 | Glutathione reductase | *Oxytricha trifallax* | EJY87957 | 3.45E-163 | 1.8.1.10 |
| Sras_6717_1 | 789 | Glutamate-cysteine ligase | *Oxytricha trifallax* | EJY69362 | 0.00 | 6.3.2.2 |
| Sopsis_11597_1 | 163 | Glutamate-cysteine ligase | *Oxytricha trifallax* | EJY69362 | 4.36E-45 | 6.3.2.2 |
| Sras_3731_1 | 174 | Glutathione synthase | *Oxytricha trifallax* | EJY75671 | 5.53E-43 | 6.3.2.3 |
| Sopsis_9287_1 | 75 | Glutathione synthase, ATP binding domain | - | PF03917.12 | 4.30E-13 | 6.3.2.3 |
| Sopsis_17091_1 | 51 | Glutathione synthase, ATP binding domain | - | PF03917.12 | 8.00E-08 | 6.3.2.3 |
| **Thioredoxin and its metabolism** | | | | | | |
| Sras_5769_1 | 264 | Thioredoxin | *Oxytricha trifallax* | EJY79260 | 8.46E-44 | - |
| Sras_11700_1 | 110 | Thioredoxin | *Oxytricha trifallax* | EJY87392 | 6.46E-22 | - |
| Sras_6724_1 | 617 | Thioredoxin family protein | *Oxytricha trifallax* | EJY84036 | 1.77E-154 | - |
| Sras_8280_1 | 304 | Thiol-disulfide isomerase-like thioredoxin | *Oxytricha trifallax* | EJY80653 | 1.01E-50 | - |
| Sras_1535_1 | 71 | Thioredoxin domain-containing protein 1 | *Oxytricha trifallax* | EJY80092 | 4.29E-14 | - |
| Sras_9186_1 | 71 | Thioredoxin domain-containing protein 1 | *Oxytricha trifallax* | EJY80092 | 4.23E-14 | - |
| Sras_4112_1 | 249 | Thioredoxin domain-containing protein 5 | *Oxytricha trifallax* | EJY77087 | 1.00E-34 | - |
| Sras_7989_1 | 286 | Thioredoxin domain-containing protein 14 | *Oxytricha trifallax* | EJY88056 | 2.95E-38 | - |
| Sras_6014_1 | 71 | Tpa_exp: thioredoxin | *Perkinsus marinus*  (Perkinsozoa) | XP_002772794 | 2.75E-08 | - |
| Sras_8783_1 | 82 | Thioredoxin-like protein 4a | *Oxytricha trifallax* | EJY79946 | 6.45E-45 | - |
| Sras_3034_1 | 105 | Thioredoxin-like isoform x2 | *Maylandia zebra* (Animal) | XP_004574214 | 8.90E-31 | - |
| Sras_4414_1 | 105 | Thioredoxin-like isoform x2 | *Maylandia zebra* | XP_004574214 | 8.77E-31 | - |
| Sopsis_411_1 | 187 | Thioredoxin | *Oxytricha trifallax* | EJY86776 | 2.62E-68 | - |
| Sopsis_4827_1 | 89 | Thioredoxin domain-containing protein | *Oxytricha trifallax* | EJY73770 | 4.54E-28 | - |
| Sopsis_8868_1 | 127 | Thioredoxin dynein outer arm protein | *Oxytricha trifallax* | EJY64419 | 3.33E-40 | - |
| Sopsis_10312_1 | 163 | Thiol-disulfide isomerase-like thioredoxin | *Oxytricha trifallax* | EJY78679 | 2.34E-51 | - |
| Sras_6414_1 | 480 | Thioredoxin reductase 1 | *Oxytricha trifallax* | EJY67312 | 0 | 1.8.1.9 |
| Sopsis_19997_1 | 489 | Thioredoxin reductase | *Oxytricha trifallax* | EJY67312 | 0 | 1.8.1.9 |
| **Metabolism of ascorbic acid** | | | | | | |
| Sras_2521_1 | 194 | Bifunctional monodehydroascorbate reductase/carbonic anhydrase nectarin-3-like | *Oxytricha trifallax* | EJY67377 | 1.39E-08 | 1.6.5.4 |
| Sras_8546_1 | 207 | Bifunctional monodehydroascorbate reductase/carbonic anhydrase nectarin-3-like | *Drosophila pseudoobscura* (Animal) | XP_001358731 | 2.25E-10 | 1.6.5.4 |
| Sras_3869_1 | 447 | Bifunctional monodehydroascorbate reductase/carbonic anhydrase nectarin-3-like | *Glarea lozoyensis*  (Fungus) | EPE36454 | 9.27E-13 | 1.6.5.4 |
| Sras_6485_1 | 225 | Bifunctional monodehydroascorbate reductase/carbonic anhydrase nectarin-3-like | *Glycine max*  (Plant) | XP_003535188 | 4.82E-08 | 1.6.5.4 |

**Table S4. Transcripts related to metabolism of non-enzymatic antioxidants in *Strombidium rassoulzadegani* (Sras) and *Strombidinopsis* sp. (Sopsis).**

EC = Enzyme Code.
